# Retrieval and Mapping of Soil Texture Based on Land Surface Diurnal Temperature Range Data from MODIS

**DOI:** 10.1371/journal.pone.0129977

**Published:** 2015-06-19

**Authors:** De-Cai Wang, Gan-Lin Zhang, Ming-Song Zhao, Xian-Zhang Pan, Yu-Guo Zhao, De-Cheng Li, Bob Macmillan

**Affiliations:** 1 State Key Laboratory of Soil and Sustainable Agriculture, Institute of Soil Science, Chinese Academy of Science, Nanjing, Jiangsu, People’s Republic of China; 2 College of Forestry, Henan Agricultural University, Zhengzhou, Henan, People’s Republic of China; 3 Institute of Surveying and Mapping, Anhui University of Science and Technology, Huainan, Anhui, People’s Republic of China; 4 ISRIC-World Soil Information, 6700, AJ, Wageningen, Netherlands; Old Dominion Univ., UNITED STATES

## Abstract

Numerous studies have investigated the direct retrieval of soil properties, including soil texture, using remotely sensed images. However, few have considered how soil properties influence dynamic changes in remote images or how soil processes affect the characteristics of the spectrum. This study investigated a new method for mapping regional soil texture based on the hypothesis that the rate of change of land surface temperature is related to soil texture, given the assumption of similar starting soil moisture conditions. The study area was a typical flat area in the Yangtze-Huai River Plain, East China. We used the widely available land surface temperature product of MODIS as the main data source. We analyzed the relationships between the content of different particle soil size fractions at the soil surface and land surface day temperature, night temperature and diurnal temperature range (DTR) during three selected time periods. These periods occurred after rainfalls and between the previous harvest and the subsequent autumn sowing in 2004, 2007 and 2008. Then, linear regression models were developed between the land surface DTR and sand (> 0.05 mm), clay (< 0.001 mm) and physical clay (< 0.01 mm) contents. The models for each day were used to estimate soil texture. The spatial distribution of soil texture from the studied area was mapped based on the model with the minimum RMSE. A validation dataset produced error estimates for the predicted maps of sand, clay and physical clay, expressed as RMSE of 10.69%, 4.57%, and 12.99%, respectively. The absolute error of the predictions is largely influenced by variations in land cover. Additionally, the maps produced by the models illustrate the natural spatial continuity of soil texture. This study demonstrates the potential for digitally mapping regional soil texture variations in flat areas using readily available MODIS data.

## Introduction

Detailed soil resource information is essential for fully satisfying the requirements of agricultural development and environmental management. Quickly and accurately obtaining such soil information is a key challenge facing soil science. As a relatively stable natural property of soil, soil texture is an important factor that influences a series of physical and chemical properties, such as soil structure, soil porosity, hydraulic properties, and nutrient retention ability. All of these factors affect soil quality. Conventional soil texture measurement methods depend on physical analyses in a laboratory, are expensive, require a large number of samples and involve a lengthy analysis to obtain the spatial distribution of soil texture over large areas. To overcome this problem, we propose the use of soil mapping and prediction based on quantitative soil-landscape models [1] and geo-statistics [2]. However, the methods based on geo-statistics [3–6] also require a large amount of measurement data. Soil information acquisition methods that utilize soil-landscape relationship theory [7–13] require several predicting factors that are difficult to obtain due to restrictions of the observing instrument. McBratney et al. [14] observed that 80% of studies on digital soil mapping in a recent 10-year period (1994–2003) used topographic indices as key predictors, while 25% of studies used vegetation as the key predictor. However, the use of topography and vegetation to estimate soil properties may not be suitable for plains and gently undulating topographic areas due to the high variability of soil properties that occur in similar topographic and vegetation conditions.

Remote sensing has become an increasingly important data source for earth science. Several researchers have attempted to demonstrate methods for estimating soil texture based on remote sensing. Some of these identify soil texture through image classification techniques using multiband remotely sensed data. Using this approach, Zhai et al. [15] identified an artificial neural network (ANN)-based method of soil texture classification based on remote sensing data from bare soils. Many other studies have explored the feasibility of estimating soil texture by establishing regression models between the reflected spectrum and percent content of sand or clay without considering the physical linkage between them [16–18]. However, only single-temporal remote sensing data were used in these studies, and few have considered how soil properties influence dynamic changes in remote images.

A large number of recent studies have shown that near-surface soil moisture can exhibit a direct linkage with remotely sensed information. In addition, methods for obtaining soil moisture content by remote sensing have become increasingly accepted [19–24]. Microwaves, thermal inertia and thermal infrared information-based remote sensing are the major data sources used to monitor soil moisture [19]. Thermal inertia is a key parameter influencing the rate of change of land surface temperature, which is closely related to soil water content. Land surface diurnal temperature range (DTR), apparent thermal inertia and real thermal inertia are the three forms of thermal inertia that can be used to estimate soil water content [20]. Mattikalli et al. [25] suggested that changes in soil moisture could be used as indicators of soil texture. The above relations illustrate that changes in microwave images and land surface temperature after a homogeneous rainfall mainly depend on the soil water content. Thus, they can be used as a proxy to identify a texture class.

Studies have been performed to estimate soil texture using microwave-based images. Santanello et al. [26] examined a straightforward method that used microwave remote sensing of near-surface soil moisture to calibrate an offline land surface model and infer soil texture and hydraulic properties at high spatial resolutions. Chang and Islam [27] and Chang et al. [28] explored the use of a multi-temporal remotely sensed brightness temperature from the Southern Great Plains in the United States. They classified soils into different textures based on the assumption that the dry-down curves of brightness temperature and soil moisture with the similar soil textures at different locations would exhibit similar behaviors. Mattikalli et al. [29] developed regression relationships for the ratio of percent sand to percent clay, which were based on brightness temperature and soil water content changes and the hypothesis that the change rates of brightness temperature and water content are related to soil texture. However, it is expensive to obtain airborne remotely sensed information. Zhu et al. [30] examined the idea that dynamic feedback patterns of the land surface, such as those captured daily by MODIS images during a short period after a major rain event, can be used to differentiate soil types. Liu et al. [31] further mapped soil texture using dynamic feedback patterns extracted from MODIS. Recently, Wang et al. [32] mapped soil texture by using fuzzy-*c*-means (FCM) clustering to obtain the changing diurnal temperature difference (from MODIS) patterns for the case of a relatively homogeneous rainfall input event. However, few studies have examined the relationship between soil texture and land surface temperature (including day temperature, night temperature and diurnal temperature range) during different periods, which correspond to different antecedent rainfall events, and estimated soil texture directly from land surface temperature.

A key objective of this study is to examine the feasibility of estimating soil texture using available MODIS land surface temperature data based on the hypothesis that the content and changes in soil water, and hence land surface temperature, are related to soil texture. This study explored a method for retrieving soil texture by studying the relationships between the content of different soil particle size fractions and land surface temperature. We then established predictive regression models and applied those for digital soil texture mapping.

## Materials and Methods

### Ethics statement

All sample sites were distributed on private land, and permission was granted by the land owner to access each site (Jinling Yang can be contacted for future permissions). The field studies did not involve endangered or protected species because all of the sample sites were located in farmland. The coordinates of sample sites ranged from 32°01′57″N to 33°10′59″N and 119°38′24″E to 120°32′20″E.

### Characterization of the area

**Fig 1:**
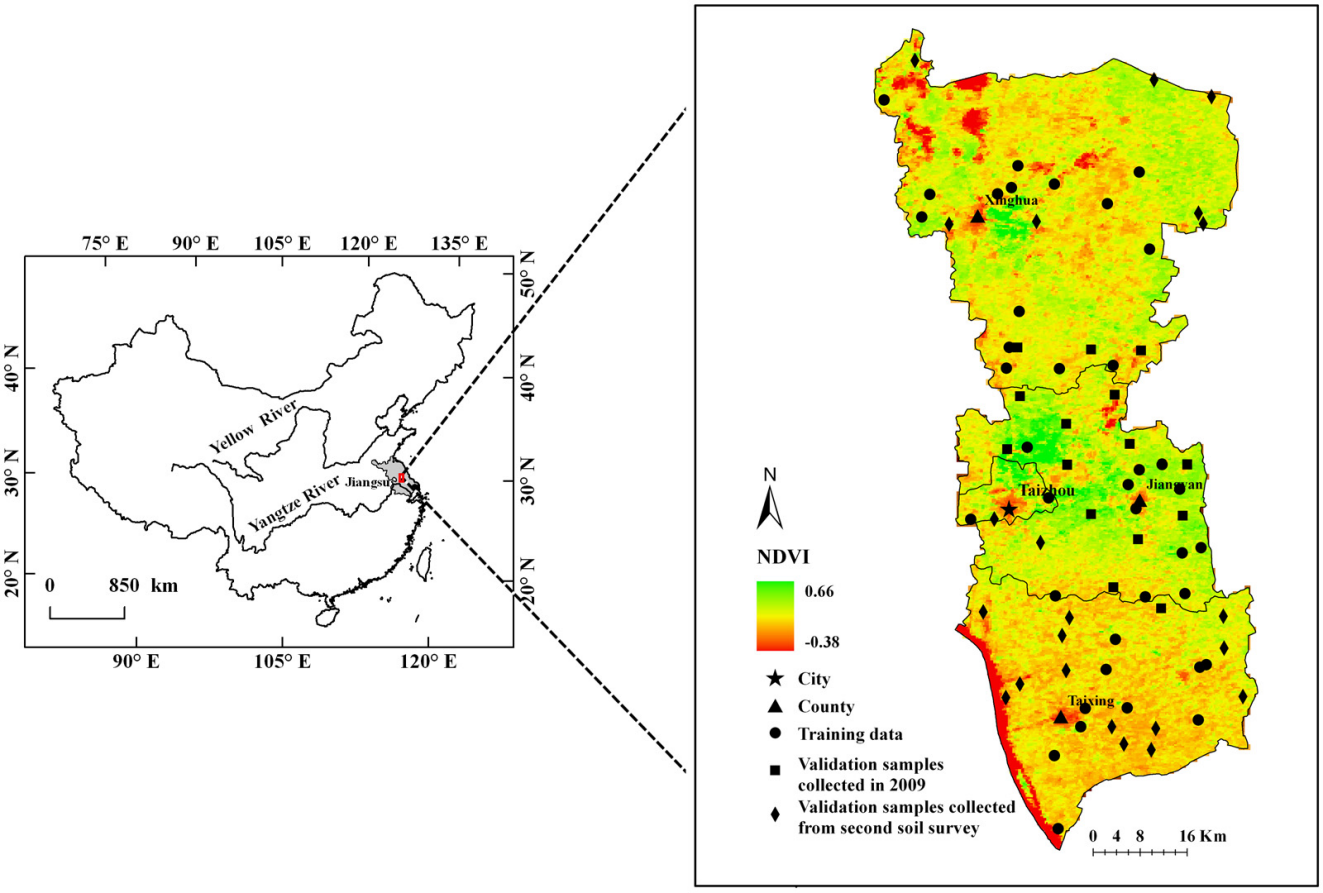
Location, sampling sites and NDVI distribution on DOY 324 in the year of 2007. NDVI: Normalized Difference Vegetation Index; DOY: Day of Year.

In selecting a study area, the variation of soil texture within the area should be significant. The area should not be too large to minimize the influence of variation related to climate. Based on the above principles, an area in the Yangtze-Huai plain, China, including Taizhou, Xinghua, Jiangyan and Taixing, was chosen as the study area (Fig 1). The 5130 km^2^ area is located in the middle of Jiangsu Province. This region was formed by alluvial deposits from the Yangtze and Huaihe Rivers. Elevations are relatively higher in the middle of the plain and lower in the northern and southern portions. The absolute elevation is typically 2–5 m in the southern areas, 5–7 m in the middle area and 1.5–5 m in the northern Lixiahe bog area. The mean annual air temperature is 14.4–15.1°C. The mean annual precipitation is 1037.7 mm. The main crops grown in the area include wheat and maize.

### Data collection

The topsoil texture data were collected from soil profile samples of the second national soil survey in Taizhou, Taixian, Xinghua and Taixing [33]. Soil texture included four fractions: sand (> 0.05 mm), coarse silt (0.01–0.05 mm), clay (< 0.001 mm) and physical clay (< 0.01 mm). The fractions were measured using the sieve-pipette method [34]. Because there is no geography coordinate data available in the 2nd soil survey data. The position of samples was determined based on the descriptions of each sampling location. Topographic and land use maps of the study area were used to collect the corresponding site attributes. A total of 62 soil samples were collected.

The daily land surface temperature data from MODIS (MOD11A1) were freely available (http://modis.gsfc.nasa.gov/data/dataprod/mod11.php). Temperature data images from MODIS were re-projected (Universal Transverse Mercator) to match the sample sites using the MODIS Reprojection Tool (MRT) software. Every sample was identified in the image, and its corresponding temperature data were extracted.

As some locations lacked remotely sensed data for one or more days, we only selected locations with daily data for all days in the study period to use for model development. Locations with partially corresponding temperature data were used as validation data. In 2009, 15 additional locations were sampled in areas of Taizhou and Jiangyan, which have both sand and clay soils, to validate the predictive results (Fig 1). The numbers of data points used as the training data were 43, 38 and 39 in 2004, 2007 and 2008, respectively.

### Selection of the study periods

One of the problems of utilizing remote sensing for soil studies is the lack of ability to reduce the effects of land cover differences. Thus, it is necessary to avoid the land cover effect by choosing appropriate study periods. In addition, there should be several dry days following the rainfall event to see how soil moisture changes with time. Therefore, we chose three study periods that correspond to different rainfall events. Each occurred between the previous harvest and the following late autumn sowing. At that time, crops in the study area had not emerged or had little coverage. Some data were not available due to the influence of cloud cover. The periods were chosen as follows: 2004.11.1–11.3, 2004.11.5–11.7; 2007.11.18–11.21, 2007.11.23 and 2008.11.9–11.11. The antecedent rainfalls from the three periods were 4.2 mm, 12.0 mm and 6.1 mm, respectively. The available land surface temperature data were MOD11A1.A2004-306,307,308,310,311,312,MOD11A1.A2007-322,323,324,325,327 and MOD11A1.A2008-314,315,316.

### Development of prediction models

The relationships between the content of different soil particle size fractions and land surface day temperature, night temperature and DTR were analyzed. Linear regression models were established between the land surface DTR and the sand (> 0.05 mm), clay (< 0.001 mm) and physical clay (< 0.01 mm) contents. The Statistical Program for Social Sciences (SPSS 13.0 for Windows) was used to conduct the analyses.

### Predictive soil texture mapping

We assumed that the variation in the change rate of DTR following a homogeneous rainfall event can be used to estimate soil texture, and the variation at a certain point may become the most significant soil texture indicator. Therefore, we used the models for each day to estimate soil texture and compared the prediction results. Maps of sand, clay and physical clay were created based on the model with the minimum Root Mean Squared Error (RMSE). Based on the predictive regression models, the spatial distribution patterns of sand, clay and physical clay were estimated for the entire study area and mapped using ArcInfo9.2.

### Validation

Values of sand, clay and physical clay predicted by the models were compared to the observed values from validation sites to assess model performance. The indicators used to evaluate the success of the predictions were R^2^, Mean Error (ME), Mean Absolute Error (MAE) and Root Mean Squared Error (RMSE).

$$ME=\frac{1}{n}\sum_{j=1}^{n}\left(P_{j}-O_{j}\right)$$ 1

$$MAE=\frac{1}{n}\sum_{j=1}^{n}\left|P_{j}-O_{j}\right|$$ 2

RMSE=[1n∑j=1n(Pj−Oj)2]12 3

where *P*_*j*_ and *O*_*j*_ are the predicted value and observed value, respectively and n is the number of observations. ME is a preferred function for estimating the general accuracy of models. The closer the value is to 0, the smaller the general deviation. MAE and RMSE are functions that estimate the accuracy and stability of models. The smaller the value, the better are the accuracy and stability of the models.

Furthermore, to illustrate the distribution of the absolute error (AE) in the study area, the interpolated maps of AE were created using the kriging method for both training samples and validation samples.

## Results and Discussion

### Relationships between the land surface day temperature and content of soil particle size fractions

Land surface day temperature responds to the water content of the topsoil. Specifically, soils with a lower water content exhibit higher land surface temperatures. Soils with higher water content have lower land surface temperatures. Table 1 shows the relationships between land surface day temperature and content of different soil particle size fractions. The content of sand shows a significant positive relationship with land surface day temperature (except DOY 2008 314–316), while the clay and physical clay contents exhibit a strong negative relationship with land surface day temperature. There is no significant relationship between the coarse silt content and land surface day temperature.

**Table 1:** Linear correlations between the land surface day temperature and content of soil particle size fractions.

DOY	Size fractions (mm)	T_d04307_	T_d04308_	T_d04310_	T_d04312_
2004(307,308,310,312)	0.01–0.05	-0.224	-.329(*)	-0.211	-0.291
	> 0.05	.443(**)	.457(**)	.528(**)	.596(**)
	< 0.001	-.354(*)	-.357(*)	-.503(**)	-.543(**)
	< 0.01	-0.346(*)	-0.280	-.456(**)	-.478(**)
2007(323–325,327)	Size fractions (mm)	T_d07323_	T_d07324_	T_d07325_	T_d07327_
	0.01–0.05	-.233	-0.131	-.408(*)	-0.306
	> 0.05	.623(**)	.637(**)	.753(**)	.661(**)
	< 0.001	-.451(*)	-.628(**)	-.505(**)	-.487(**)
	< 0.01	-.601(**)	-.706(**)	-.616(**)	-.587(**)
2008(314–316)	Size fractions (mm)	T_d08314_	T_d08315_	T_d08316_	
	0.01–0.05	0.130	0.020	0.010	
	> 0.05	0.180	0.140	0.310	
	< 0.001	-0.241	-0.189	-.351(*)	
	< 0.01	-0.156	-0.165	-.364(*)	

DOY = day of year; Td = day temperature;

*,**significant at p < 0.05, p < 0.01, respectively.

Following a rainfall event, and assuming constant climate conditions, the soil water content is expected to vary based on differences in soil structure and water holding capacity, which in turn depend on the soil texture [29]. During the dry-down period, a sandy soil with a lower water holding capability is expected to have a faster depletion rate and a lower soil water content, leading to a higher land surface temperature. A clay soil with a higher water holding capability will have a slower depletion rate and higher soil water content, resulting in a lower land surface temperature. Consequently, we observe a close relationship between land surface day temperature and soil texture.

### Relationships between the land surface night temperature and content of soil particle size fractions

Table 2 shows the relationships between the land surface night temperature and content of different soil particle size fractions. There appears to be a significant negative relationship between the sand content and land surface night temperature. A significant positive relationship exists between clay and physical clay contents and land surface night temperature. There is no significant relationship between the coarse silt content and land surface night temperature. As the land surface is no longer receiving heat at night, evenings result in a cooling process. Soil thermal inertia, one of the soil thermal characteristics, is closely related to soil water content, which in turn influences changes in land surface temperature. After a rainfall event, a sandy soil with a lower water holding capability will have a faster depletion rate and lower soil water content. Hence, the sandy soil has a smaller thermal inertia, which leads to a faster heat depletion rate and lower land surface night temperature. A clay soil with a higher water holding capability will have a slower depletion rate and higher soil water content. Therefore, the clay has a larger thermal inertia, which leads to a slower heat depletion rate and higher land surface night temperature.

**Table 2:** Linear correlations between the land surface night temperature and content of soil particle size fractions.

DOY	Size fractions(mm)	T_n04306_	T_n04307_	T_n04308_	T_n04310_	T_n04311_	T_n04312_
2004(306–308,310–312)	0.01–0.05	0.09	0.078	0.142	-0.244	-0.134	0.126
	> 0.05	-.610(**)	-.665(**)	-.568(**)	-0.224	-.550(**)	-.632(**)
	< 0.001	.594(**)	.653(**)	.546(**)	0.221	.580(**)	.615(**)
	< 0.01	.656(**)	.731(**)	.566(**)	.465(*)	.758(**)	.645(**)
2007(322–325,327)	Size fractions(mm)	T_n07322_	T_n07323_	T_n07324_	T_n07325_	T_n07327_	
	0.01–0.05	0.053	0.093	0.158	.425(*)	0.161	
	> 0.05	-.545(**)	-.455(**)	-.499(**)	-.620(**)	-.463(**)	
	< 0.001	.505(**)	.484(**)	0.352(*)	.416(*)	.428(**)	
	< 0.01	.655(**)	.500(**)	0.498(**)	.423(*)	.451(**)	
2008(314–316)	Size fractions (mm)	T_n08314_	T_n08315_	T_n08316_			
	0.01–0.05	0.09	0.10	0.21			
	> 0.05	-.418(*)	-.623(**)	-.439(**)			
	< 0.001	.374(*)	.543(**)	.533(**)			
	< 0.01	.528(**)	.756(**)	.654(**)			

DOY = day of year; Tn = night temperature;

*,**significant at p < 0.05, p < 0.01, respectively.

### Relationships between the land surface DTR and content of soil particle size fractions

Table 3 shows the relationships between the land surface DTR and content of different soil particle size fractions. There is a positive relationship between the sand content and land surface DTR. Conversely, there is a negative relationship between the clay and physical clay contents and land surface DTR. There is no significant correlation between the coarse silt content and land surface DTR. This is expected because thermal inertia, which is a function of the soil water content, mineral content and organic composition, controls land surface DTR. Therefore, soils with higher clay contents often hold more water and promote soil organic matter accumulation [35]. After a rain, a sandy soil will have a lower soil water content and higher land surface DTR than a more clayey soil.

**Table 3:** Linear correlations between the land surface diurnal temperature range and content of soil particle size fractions.

DOY	Size fractions (mm)	T_04307_	T_04308_	T_04310_	T_04312_
2004(307,308,310,312)	0.01–0.05	-0.147	-0.243	-0.082	-0.243
	> 0.05	.622(**)	.565(**)	.609(**)	.667(**)
	< 0.001	-.577(**)	-.505(**)	-.584(**)	-.625(**)
	< 0.01	-.623(**)	-.481(*)	-.658(**)	-.598(**)
2007(323–325,327)	Size fractions (mm)	T_07323_	T_07324_	T_07325_	T_07327_
	0.01–0.05	-0.210	-0.157	-.461(**)	-0.265
	> 0.05	.696(**)	.585(**)	.728(**)	.640(**)
	< 0.001	-.606(**)	-0.484(**)	-.489(**)	-.523(**)
	< 0.01	-.712(**)	-.612(**)	-.533(**)	-.592(**)
2008(314–316)	Size fractions (mm)	T_08314_	T_08315_	T_08316_	
	0.01–0.05	0.044	0.068	0.131	
	> 0.05	.435(**)	.554(**)	.431(**)	
	< 0.001	-.414(*)	-.521(**)	-.510(**)	
	< 0.01	-.528(**)	-.668(**)	-.595(**)	

DOY = day of year; T = diurnal temperature range;

*,**significant at p < 0.05, p < 0.01, respectively.

**Fig 2:**
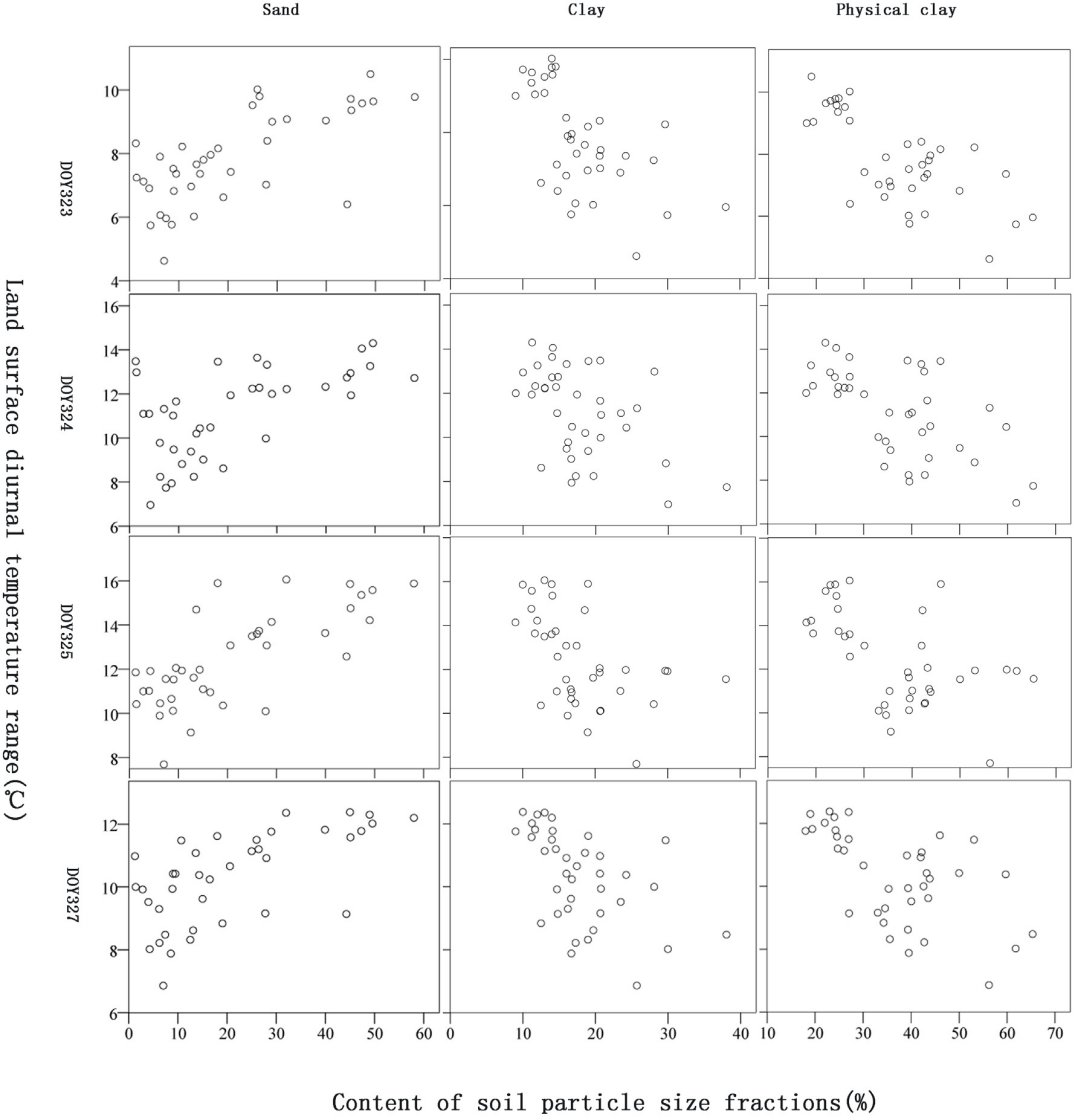
Scatter plots of DTR value and content of soil particle size fractions on DOY 323–325,327 in the year of 2007. For respective correlation coefficients refer to Table 3. DOY: Day of Year.

Overall, we see significant relationships between land surface day temperature, night temperature and DTR with sand, clay and physical clay contents. The relationships between the land surface DTR and sand, clay and physical clay contents are the strongest. Of these, the relationships in 2007 with the maximum rainfall input (12 mm) exhibit the best correlation (all significant at p<0.01 level), and the scatter plots of content of soil particle size fractions vs. DTR value (Fig 2) show diffuse but highly-significant linear correlations. This suggests that the antecedent rainfall of the study period was sufficient for analyzing the water content variations and corresponding changes in the soil surface moisture of different soil texture classes. Although the relationships are significant, there is no clear regularity as the number of days after a rainfall increases. We theorize that the rainfall events were not intense enough, resulting in a very low soil surface water content after the first two days. In addition, the thermal properties of different soil textures are also different [35]. Therefore, two soils with similar water contents may have different land surface DTRs. More simulation experiments are needed to clarify the relationship between soil water content and land surface temperature for different soil texture classes. The relationships between the land surface day temperature and night temperature and sand, clay and physical clay contents were not always significant. This may be because the land surface day and night temperatures are not always sufficient indicators of soil water content. Consequently, this study used land surface DTR to estimate sand, clay and physical clay contents.

### Retrieval and mapping of soil texture based on land surface DTR

Based on an analysis of the relationships between the land surface day temperature, night temperature, DTR and content of different soil particle size fractions, we selected the land surface DTR from 2007 for further soil texture analysis. It was chosen because it exhibited the strongest relationship with soil texture. The results show that the sand, clay and physical clay contents exhibit close linear regression relationships with land surface DTR (Tables 4 and 5). The tables show that the regression relationships vary daily. We used the daily models to estimate soil texture. We then compared the prediction results.

**Table 4:** Predictive models of sand content on different days

DOY (2007)	Predictive models	R^2^
323	Sand = 7.7408×T_07323_-39.664	0.48
324	Sand = 4.7151×T_07324_-31.417	0.34
325	Sand = 5.4945×T_07325_-47.293	0.53
327	Sand = 7.0039×T_07327_-51.152	0.41

DOY = day of year; T = diurnal temperature range.

**Table 5:** Predictive models of clay and physical clay contents on different days.

DOY(2007)	Clay		Physical clay	
	Predictive models	R^2^	Predictive models	R^2^
323	Clay = -2.6113×T_07323_+38.418	0.37	Physical clay = -6.0875×T_07323_+84.604	0.51
324	Clay = -1.5115×T_07324_+34.755	0.23	Physical clay = -3.7913×T_07324_+79.047	0.37
325	Clay = -1.4297×T_07325_+35.715	0.24	Physical clay = -3.0943×T_07325_+75.331	0.28
327	Clay = -2.2217×T_07327_+40.839	0.27	Physical clay = -4.9842×T_07327_+88.234	0.35

DOY = day of year; T = diurnal temperature range.

Independent locations were available for validation. The numbers of data points used in the validation dataset were 33 on DOY 323, 36 on DOY 324, 20 on DOY 25 and 39 on DOY 27. Table 6 displays the results of the evaluation of the predictive results of sand, clay and physical clay contents. The maximum R^2^ values for sand, clay and physical clay contents are 0.45, 0.35 and 0.36, respectively. The minimum RMSEs are 10.69%, 4.57% and 10.94%, respectively. The minimum MAEs and MEs are 8.72%, 3.44% and 8.87% and 2.76%, 0.10% and -2.93%, respectively. According to the validation results, the model with the highest R^2^ value may not always provide the best prediction. That is because R^2^ only indicates the degree of fit of the model. However, a good prediction should also have a small prediction RMSE.

**Table 6:** Evaluation of prediction results of sand, clay and physical clay contents.

DOY(2007)	R^2^			ME (%)			MAE (%)			RMSE (%)		
	Sand	Clay	Physical clay	Sand	Clay	Physical clay	Sand	Clay	Physical clay	Sand	Clay	Physical clay
323(N = 33)	0.37	0.26	0.33	3.78	0.10	-5.93	9.63	4.36	11.31	12.51	5.27	13.38
324(N = 36)	0.32	0.29	0.29	4.59	0.70	-6.09	8.72	3.44	10.23	10.69	4.57	12.99
325(N = 20)	0.45	0.10	0.26	11.10	2.97	-6.27	11.98	5.74	8.87	15.48	6.53	10.94
327(N = 39)	0.37	0.35	0.36	2.76	1.23	-2.93	8.90	4.08	10.71	11.31	4.97	13.37

DOY = day of year. N = number of validation data.

Thompson et al. [12] estimated surface sand content in the eastern, central and western parts of Kentucky based on a soil-landscape model that used a high resolution DEM. The R values of these predicted values, compared to observed values, were 0.82, 0.54, and 0.61, respectively. The MEs were -0.51%, -6.83% and -0.91%, respectively. The RMSEs were 1.92%, 12.34% and 3.64%, respectively. Table 6 illustrates that the ME and RMSE in this study are much larger than those from Thompson et al. However, their method was based on a soil-landscape model, which is not applicable over areas of low relief. Dematte et al. [16] established regression models between the remote sensing spectrum from high resolution remote sensing data, ETM+/LANDSAT-7, and sand and clay contents, to estimate soil surface sand and clay contents. Their R^2^ values of the predicted values, which were plotted against observed values, were 0.50 and 0.61, which are higher than this study. However, Maselli et al. [17] estimated the soil surface sand content using the static spectrum from TM images over the entire Grosseto Province, where soil texture showed a wide variety. Their R^2^ and RMSE were 0.10 and 18.7%, respectively. The accuracy of predictions is often affected by the spatial heterogeneity and the complexity of landscape conditions. Comparisons of these statistics can give a qualitative estimate of the predictive accuracy.

**Fig 3:**
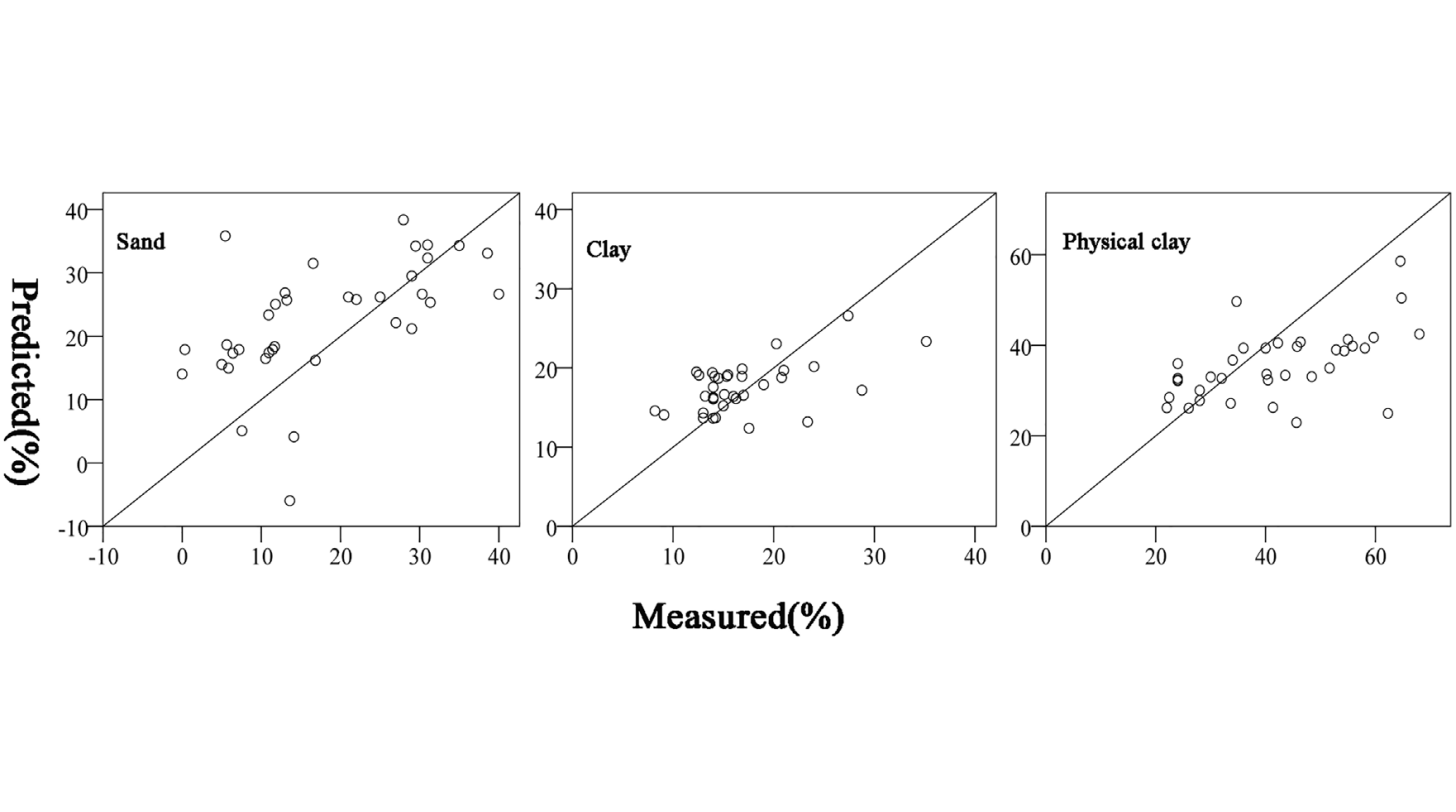
Calibration and validation scatter plots of predictive models of DOY324 in the year of 2007. For respective performance indicators refer to Table 6. DOY: Day of Year.

**Fig 4:**
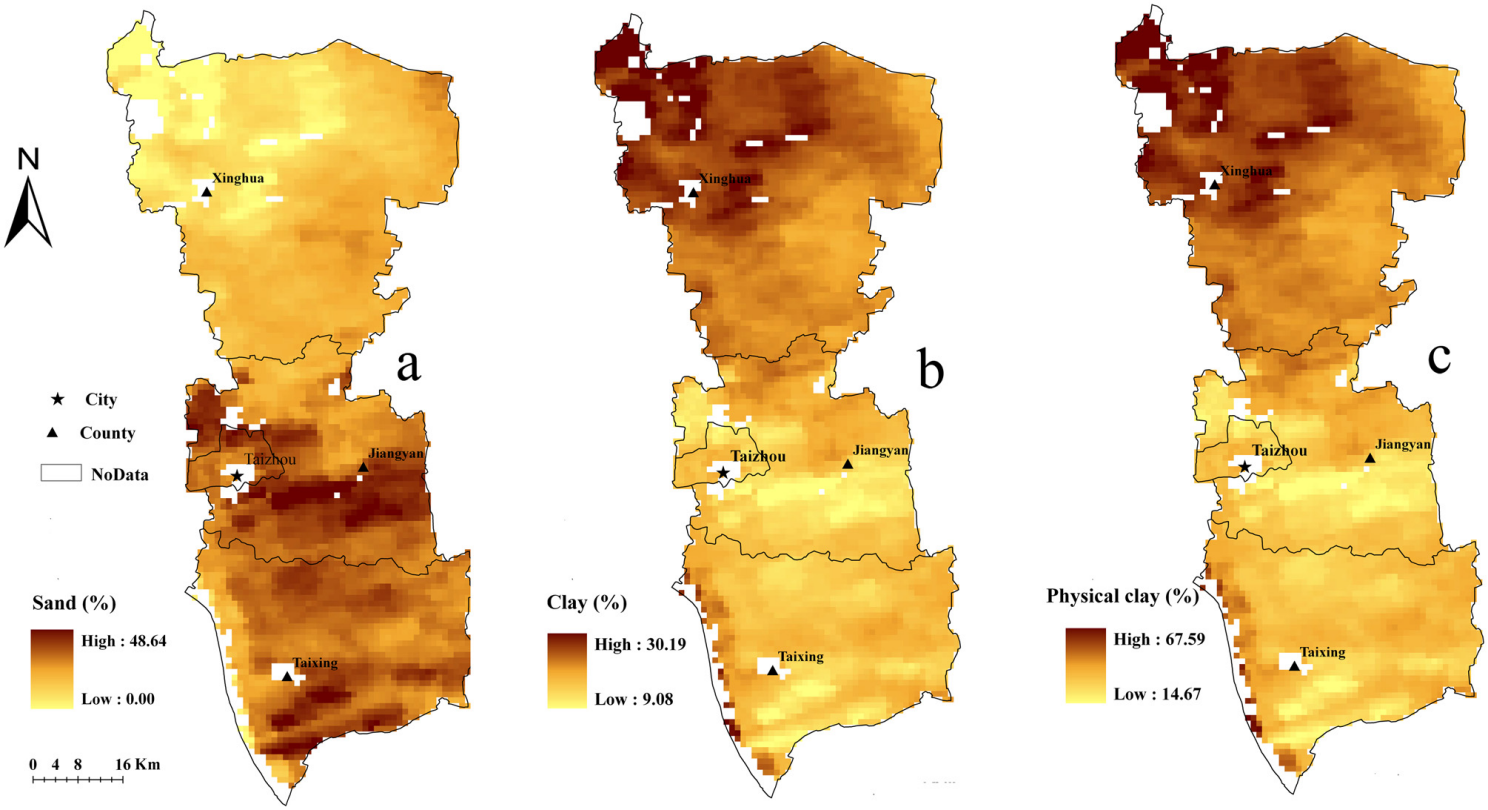
Map of the distribution of sand content (a), clay content (b) and physical clay content (c) based on the prediction.

Maps of sand and clay were generated based on the models developed for DOY324, which had the minimum RMSE. Because significant data are missing from the DOY325 MODIS image, the map of physical clay content was also created using the model developed for DOY 324. Calibration and validation scatter plots for the maps created using the model developed for DOY 324 were shown in Fig 3. The coarse silt content map was not mapped based on the model because there is no significant correlation between coarse silt content and land surface DTR. However, the coarse silt content map can be obtained by the raster calculator, using (coarse silt(%) = 100%-sand(%)—physical clay(%)). The map of the distribution of sand content, clay content and physical clay content illustrate that the sand content gradually increases from north to south, while the clay and physical clay contents exhibit the opposite trend. In general, the soil texture is clay in the Lixiahe bog area, while the soil close to the Yangtze River is predominantly sandy (Fig 4). According to the second national soil survey, from the north bank of the Yangtze River to the southern margin of the Lixiahe bog area, where the parent material is mainly the alluvial material of the Yangtze River, the soils are normally a sandy loam texture. On the contrary, the parent material in the Lixiahe bog area is mainly lacustrine sediments and the soil texture is clay [33]. This observation is consistent with the results of the second national soil survey. Additionally, the maps produced by the models maintain a soil property spatial continuity pattern better than most traditional soil maps.

**Fig 5:**
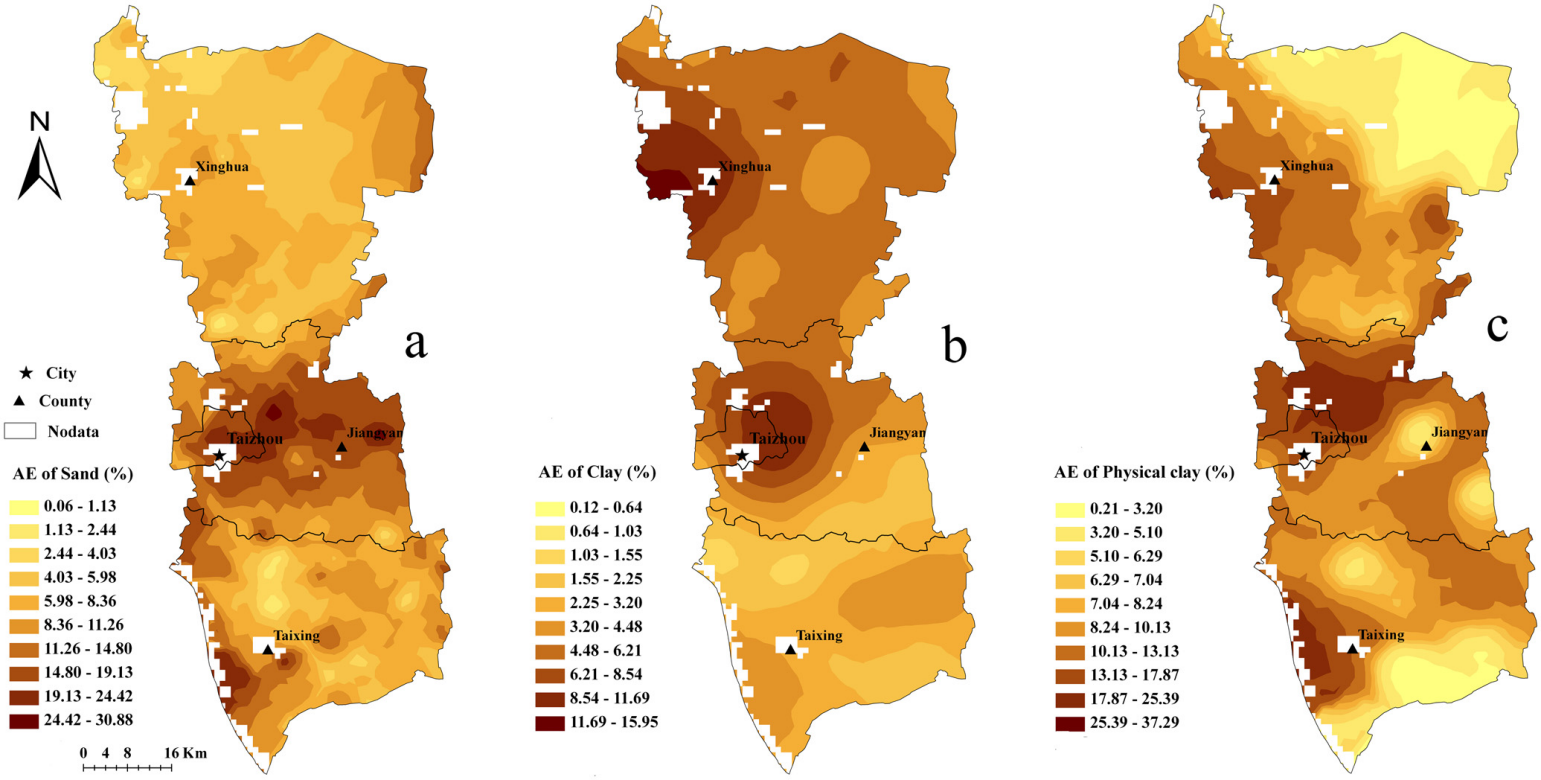
Map of the predictive AE distribution of sand content (a), clay content (b) and physical clay content (c). AE: Absolute Error.

To illustrate the distribution of the AEs in the study area, the AEs of both the training and validation datasets were interpolated using the ordinary kriging method. The maps of the predictive AE distribution (Fig 5) show that areas of higher AEs coincide with similar areas of higher NDVI values, especially for clay and physical clay (Fig 1). This is interpreted to mean that vegetation cover significantly influences the predictive results. Additionally, the areas covered by water (NDVI < 0 in Fig 1) also show a higher AE because large areas of water strongly influence land surface temperature.

This study proposed a simple linear regression model based on the DTR available from MODIS archives to obtain soil texture with adequate accuracy. The method requires a rainfall event that saturates the soil of the study area homogeneously. Therefore, large areas should be divided into different sub-areas according to rainfall features, and independent models for each rainfall zone should be established to obtain soil texture distribution over large areas. The method is based on the assumption that the surface condition is bare soil. Although we chose the periods between the previous harvest and the subsequent autumn sowing as the study periods, the accuracy of the predictive maps was still largely influenced by vegetation. In addition, the resolution of DTR data from MODIS is relatively low (1 km), the low purity of the mixed pixels is of particularly concern, which may lead to weak correlations of the predicted versus measured (Fig 3). More studies should be done to reduce the effects of land cover differences. With the development of remote sensing technology, more high spatial and temporal resolution remote sensing data will be freely available to obtain DTR with high accuracy, which could greatly enhance the prediction accuracy of the model, and broaden its applications.

## Conclusions

Land surface DTR has a strong relationship with sand, clay and physical clay contents. Linear regression models of land surface DTR and sand, clay and physical clay contents were developed. In addition, maps of sand, clay and physical clay were generated based on the models with the minimum RMSE. Validation results suggest that the models can estimate soil texture with adequate accuracy and stability. Therefore, the method used in this study has significant potential for improving the digital mapping of soil properties in low relief areas. However, the method is based on the assumption that the surface condition is bare soil, which is particularly applicable in intensively cultivated alluvial plains with clear bare-soil periods.
